# Supplementary material for: Neural heterogeneity promotes robust learning
Source: Nat Commun. 2021 Oct 4;12:5791. doi: 10.1038/s41467-021-26022-3 (PMC8490404; doi:10.1038/s41467-021-26022-3)
Supplement: Supplementary file 1 — Supplementary Information [file 41467_2021_26022_MOESM1_ESM.pdf]

Supplementary Information for Neural heterogeneity promotes robust learning

1 Supplementary Tables **2**

1.1 Heterogeneity on other spiking neuron hyperparameters 2

2 Supplementary Figures **3**

2.1 Membrane time constant distribution consistency across trials 3

2.2 Synaptic time constant distribution consistency across trials 4

2.3 Confusion matrices DVS-gesture dataset 5

2.4 Grid search of parameters 6

2.5 All trials Fashion-MNIST 6

2.6 Robustness under other time constant distributions 7

2.7 Visualisation of temporal structure of DVS128 gesture dataset samples 8

2.8 Membrane time constant distribution learned from different initialisation 9

2.9 Synaptic time constant distribution learned from different initialisation 10

2.10 Membrane time constant fitted to Gamma and Log-normal distributions 11

2.11 Synaptic time constant fitted to Gamma and Log-normal distributions 12

2.12 Experimentally observed time constant fitted to Gamma and Log-normal distributions 13

2.13 Performance on Augmented SHD dataset 14

Supplementary References **15**

1 Supplementary Tables

1.1 Heterogeneity on other spiking neuron hyperparameters

	**HomInit-StdTr** τ_m and τ_s			**HetInit-HetTr** τ_m and τ_s		
	U_{th}	U_0	U_r	U_{th}	U_0	U_r
**HomInit-StdTr**	72.8 ± 3.4	72.8 ± 3.4	72.8 ± 3.4	78.9 ± 2.0	78.9 ± 2.0	78.9 ± 2.0
**HetInit-StdTr**	71.1 ± 2.3	69.9 ± 2.3	70.8 ± 3.3	79.2 ± 2.9	74.8 ± 4.9	79.1 ± 2.7
**HomInit-HetTr**	73.7 ± 2.8	71.6 ± 3.1	70.5 ± 3.7	79.1 ± 1.8	76.1 ± 3.3	76.4 ± 2.7
**HetInit-HetTr**	73.2 ± 2.8	72.0 ± 2.6	69.4 ± 3.8	79.2 ± 2.8	74.9 ± 6.6	75.4 ± 6.6

Supplementary Table 1: Performance comparison among different RSNN configurations on SHD testing set. The configuration for τ_m and τ_s was used as specified in the top row. Initialisation and training schemes were applied only for the parameter in its corresponding column

2 Supplementary Figures

2.1 Membrane time constant distribution consistency across trials

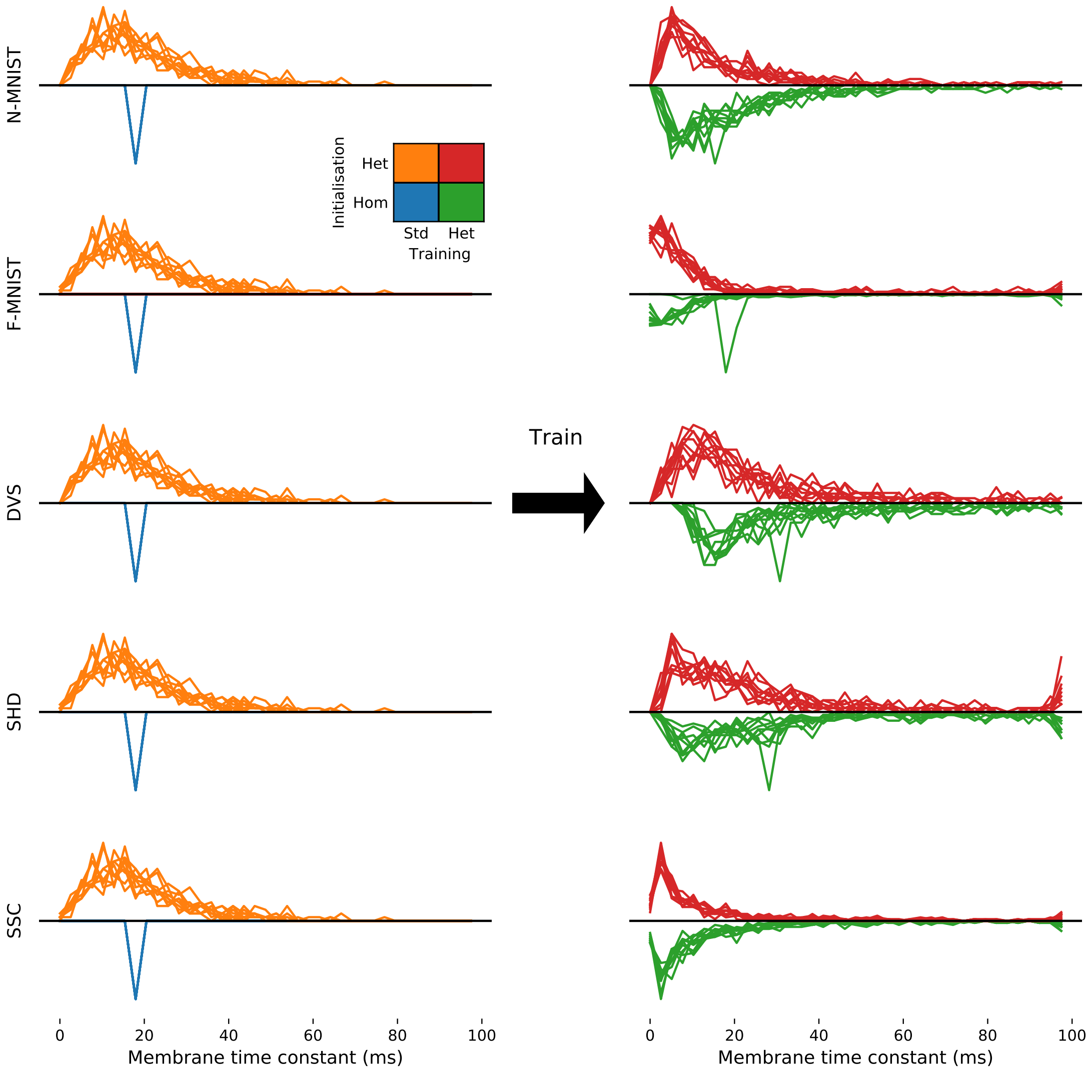

Supplementary Figure 1: Breakdown of membrane time constant distribution for each trial for consistency checking on each dataset.

2.2 Synaptic time constant distribution consistency across trials

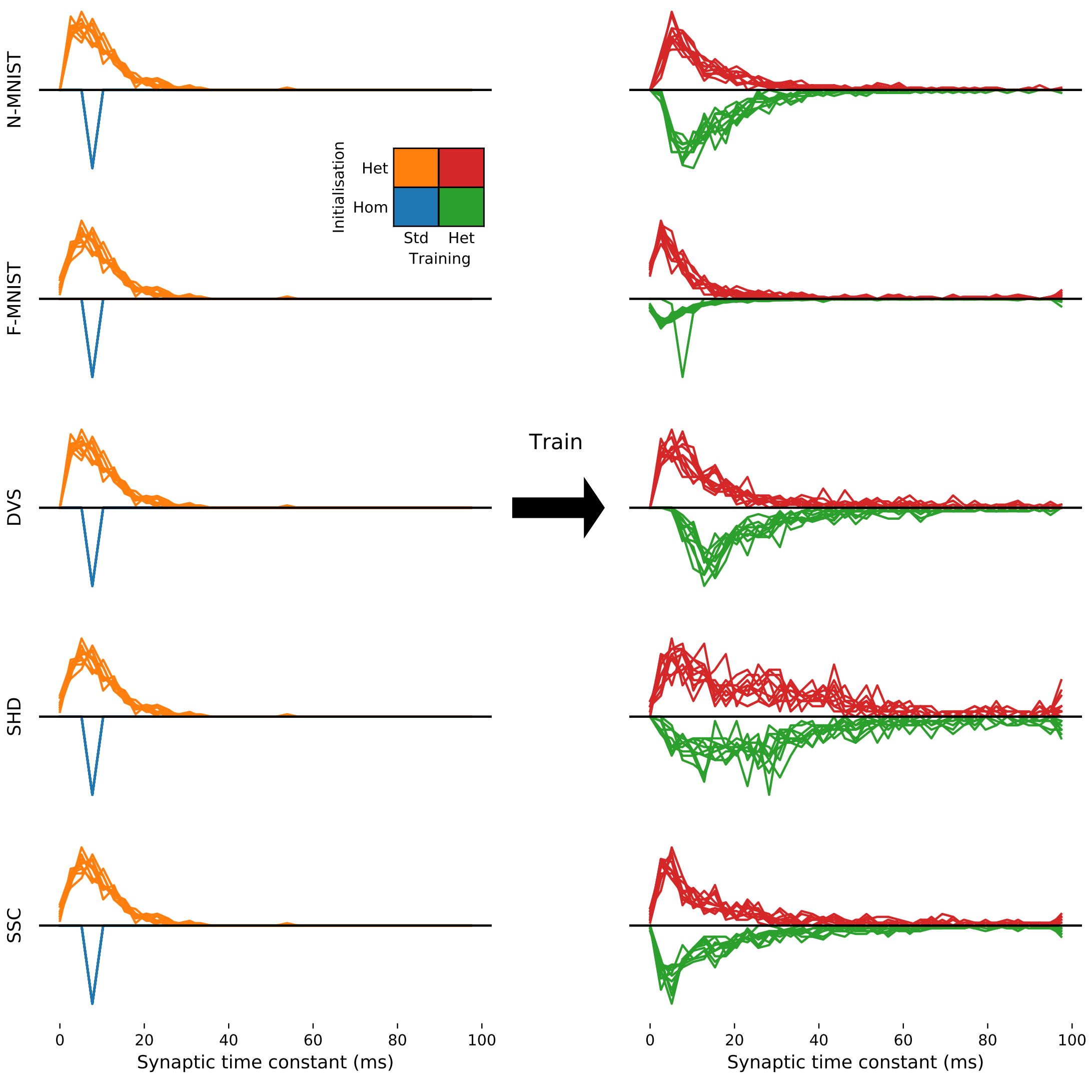

Supplementary Figure 2: Breakdown of synaptic time constant distribution for each trial for consistency checking on each dataset.

2.3 Confusion matrices DVS-gesture dataset

The classes correspond to

1. Hand clapping
2. Right hand wave
3. Left hand wave
4. Right arm clockwise
5. Right arm counter-clockwise
6. Left arm clockwise
7. Left arm counter-clockwise
8. Arm roll
9. Air drum
10. Air guitar

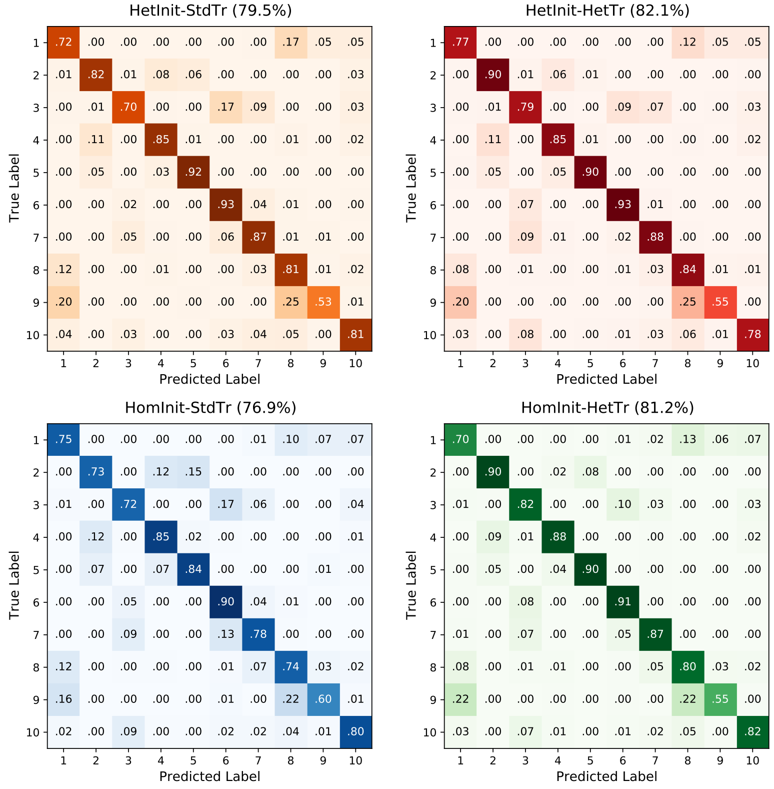

Supplementary Figure 3: Full confusion matrix DVS dataset for each configuration

2.4 Grid search of parameters

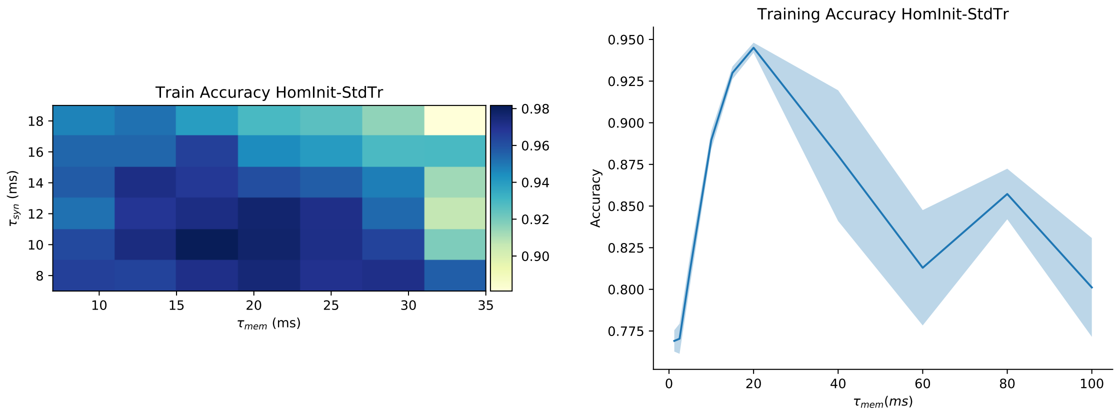

Supplementary Figure 4: A. Grid search of of optimal membrane and synaptic time constants on the SHD dataset for a single trial **B.** Grid search of of optimal membrane time constant for the SHD dataset given synaptic time constant is 10ms for 10 trials. Result $\tau_{syn} = 10ms, \tau_{mem} = 20ms$ which is the same as those time constants used in [1]

2.5 All trials Fashion-MNIST

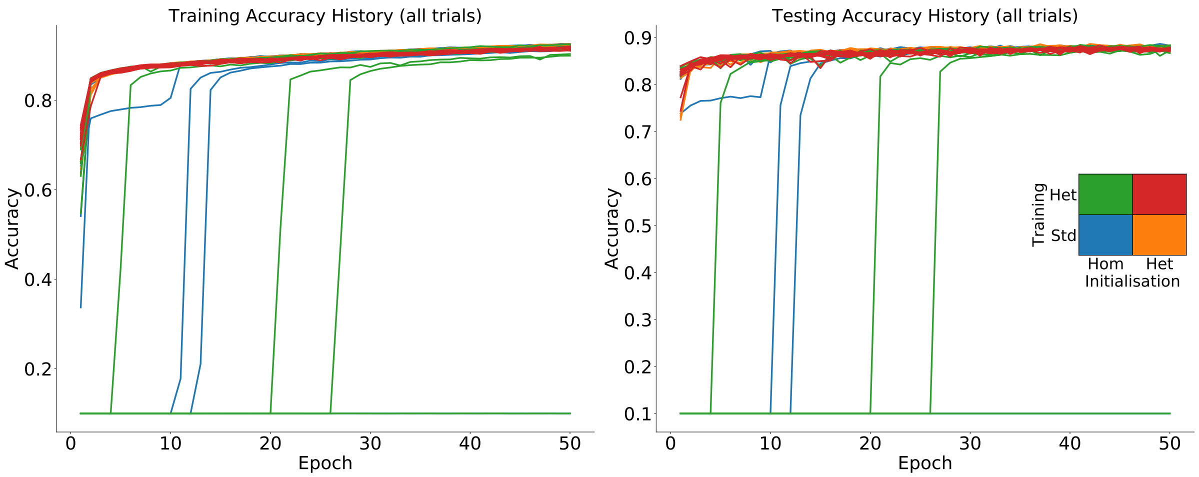

Supplementary Figure 5: All individual trials under all four configurations (total of 40). Note how a heterogeneous initialisation results in faster convergence consistently.

2.6 Robustness under other time constant distributions

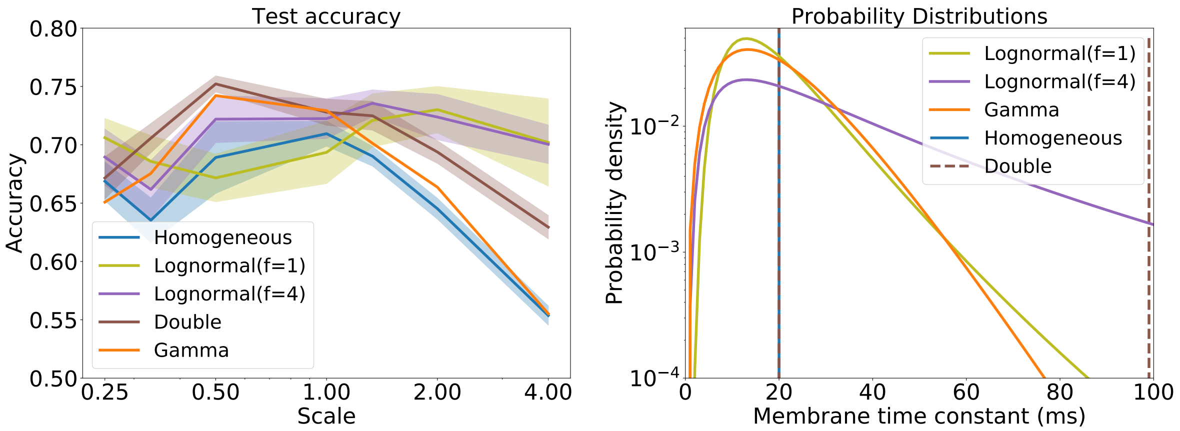

Supplementary Figure 6: Left: Test accuracy with standard training and different heterogeneous initialisations for both membrane and synaptic time constants **Right**: Probability distributions for the membrane time constants (Homogeneous, Lognormal, Double (like homogeneous but 5% of the membrane and synaptic time constants are $100ms$) and Gamma)

2.7 Visualisation of temporal structure of DVS128 gesture dataset samples

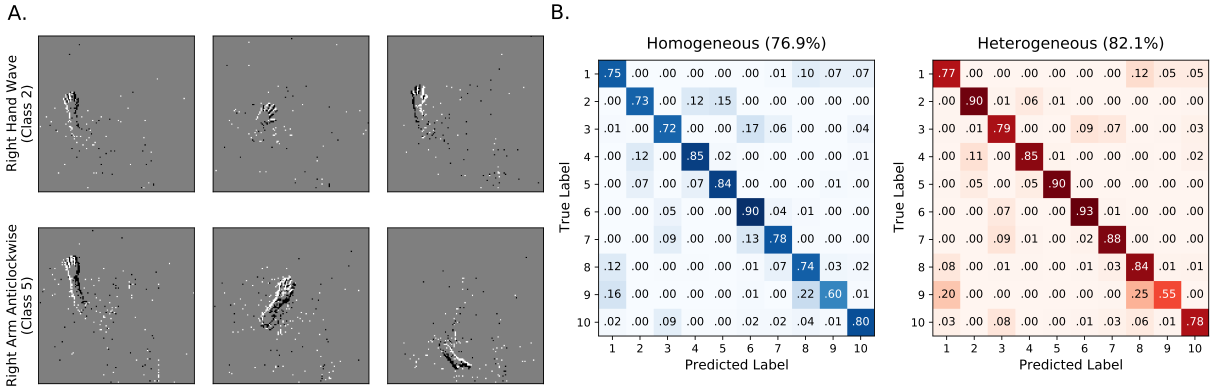

Supplementary Figure 7: A. Visualization of two samples of the DVS128 gesture dataset. Each frame spans a 5ms window with 200ms between frames. To distinguish these gestures we need to integrate over tens of milliseconds. **B.** Confusion matrices of the DVS128 gesture dataset under the fully homogeneous (left) and fully heterogeneous (right) configurations. Class 2 (*Right hand wave*) is often incorrectly classified as 4 (*Right hand clockwise*) and 5 (*Right hand counter clockwise*) under the homogeneous configuration but not under the heterogeneous one.

2.8 Membrane time constant distribution learned from different initialisation

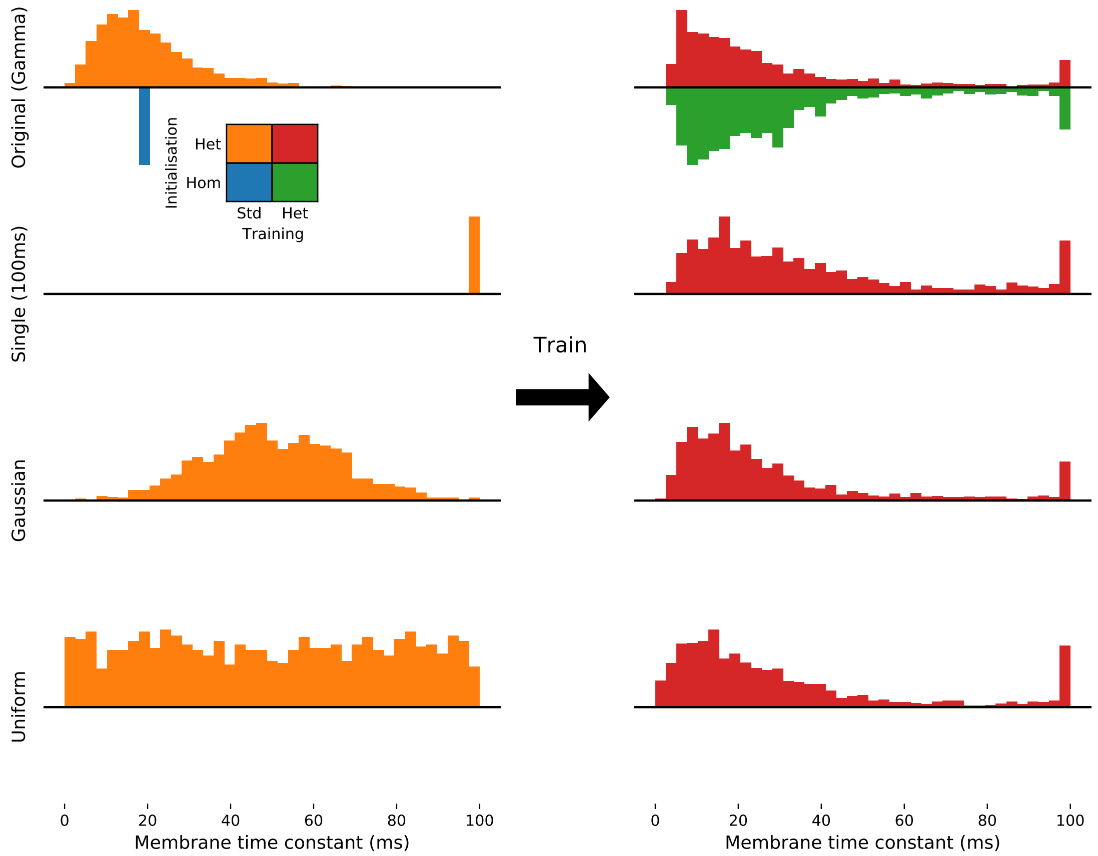

Supplementary Figure 8: Membrane time constant distributions before (left) and after (right) training for SHD dataset. Each row represents a different initialisation of the time constants.

2.9 Synaptic time constant distribution learned from different initialisation

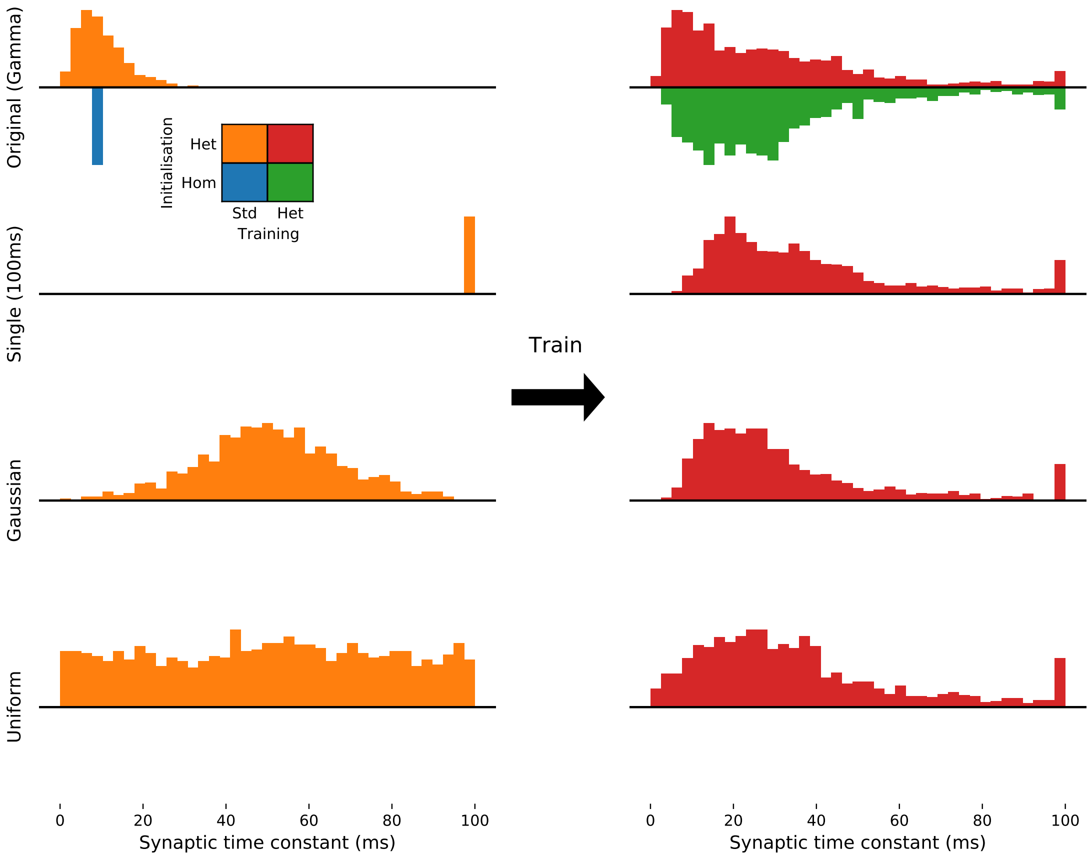

Supplementary Figure 9: Synaptic time constant distributions before (left) and after (right) training for SHD dataset. Each row represents a different initialisation of the time constants.

2.10 Membrane time constant fitted to Gamma and Log-normal distributions

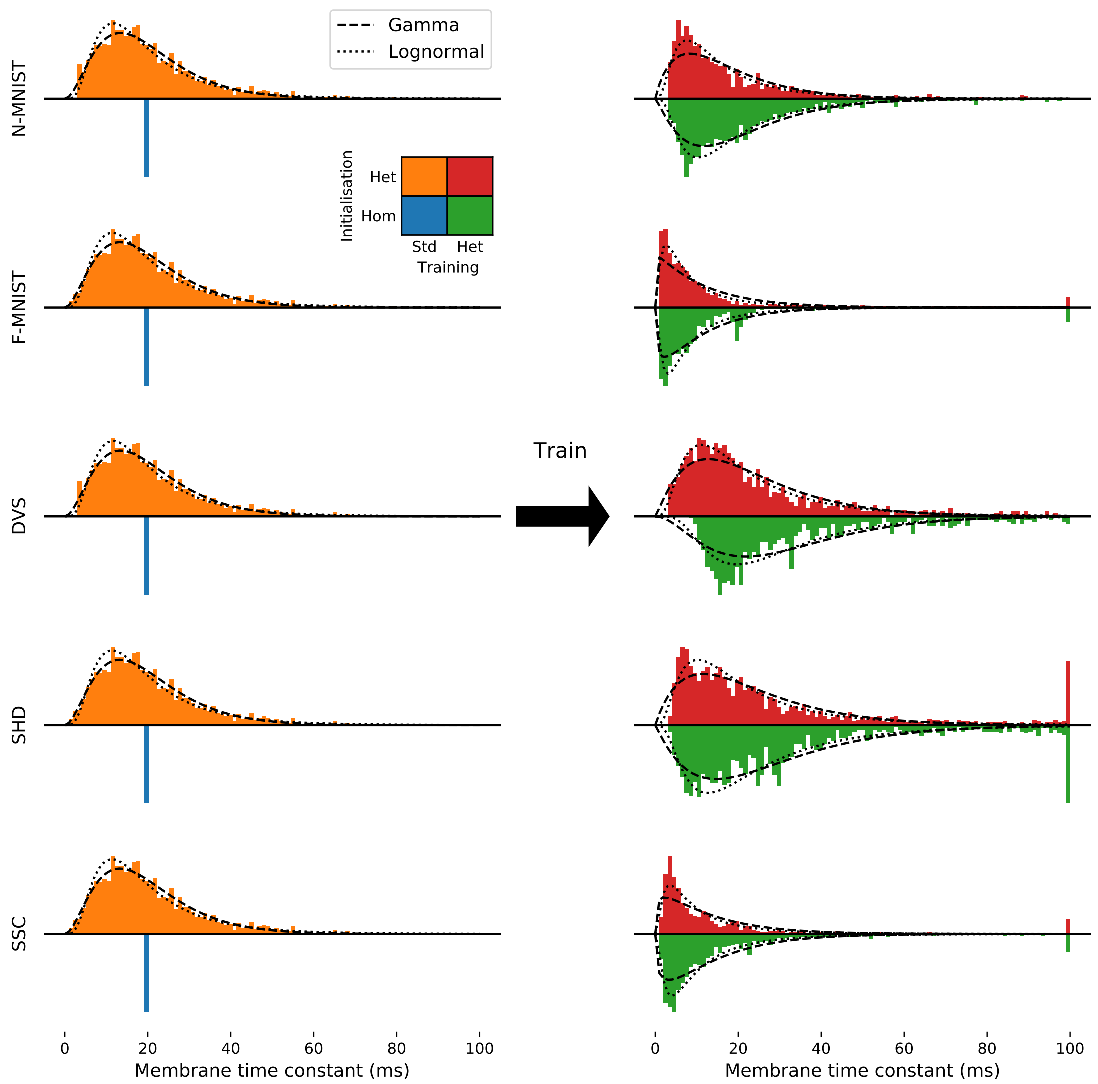

Supplementary Figure 10: Membrane time constant distributions before (left) and after (right) training for each dataset. The dashed and the dotted line represents the fitted Gamma and Log-normal distribution respectively.

2.11 Synaptic time constant fitted to Gamma and Log-normal distributions

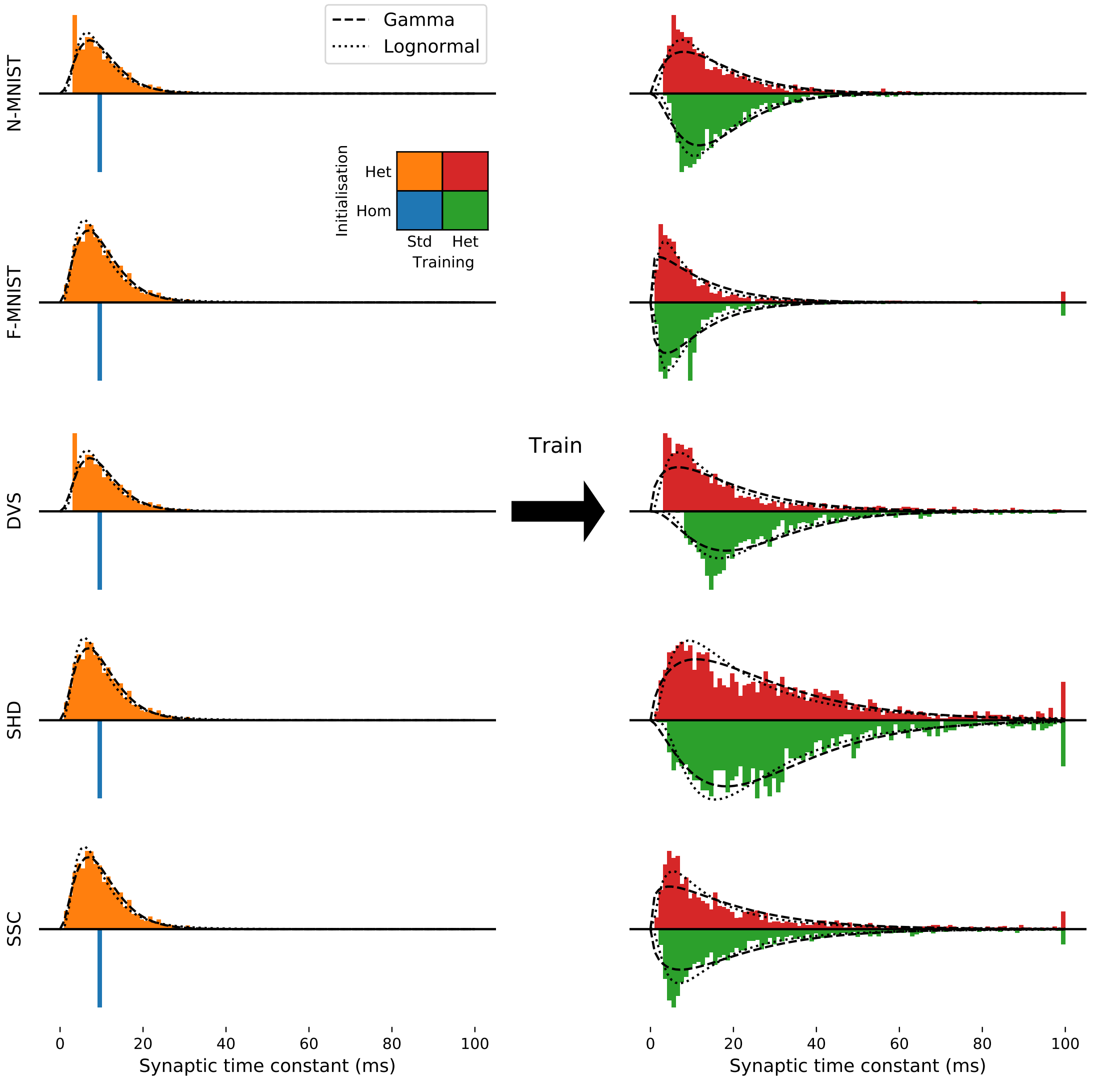

Supplementary Figure 11: Synaptic time constant distributions before (left) and after (right) training for each dataset. The dashed and the dotted line represents the fitted Gamma and Log-normal distribution respectively.

2.12 Experimentally observed time constant fitted to Gamma and Log-normal distributions

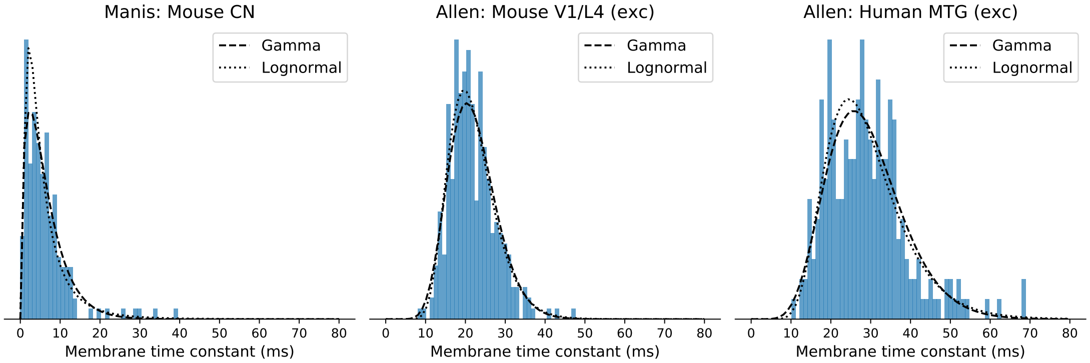

Supplementary Figure 12: Experimentally observed distributions of time constants for (left to right): mouse cochlear nucleus, multiple cell types (172 cells)[2, 3]; mouse V1 layer 4, spiny (putatively excitatory) cells (164 cells)[4, 5]; human middle temporal gyrus, spiny cells (236 cells)[4, 5]. The dashed and the dotted line represents the fitted Gamma and Log-normal distribution respectively.

2.13 Performance on Augmented SHD dataset

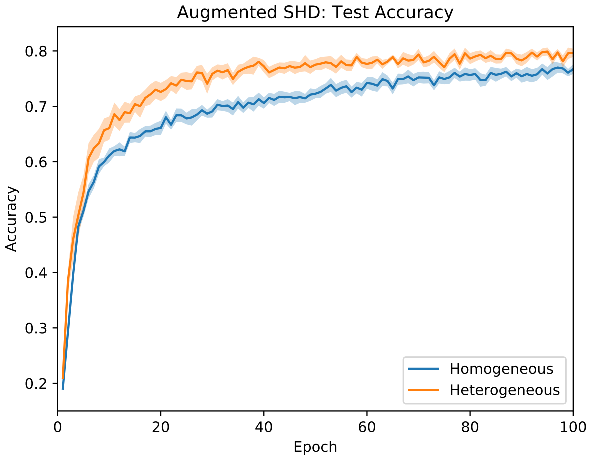

Supplementary Figure 13: Test accuracy on augmented SHD dataset consisting of channel compression and channel index noise injection as described in [6], namely adding an spike jitter to the channels by adding a random number $u \sim \mathcal{N}(0, 20)$ rounded to the nearest integer to the neuron index of every input spike and then merging every 10 neighbouring input channels. Training took place on a homogeneous-training and homogeneous-initialisation configuration (in blue) and on a heterogeneous-training homogeneous-initialisation configuration (in orange).

Supplementary References

1. Neftci, E. O., Mostafa, H. & Zenke, F. Surrogate Gradient Learning in Spiking Neural Networks: Bringing the Power of Gradient-Based Optimization to Spiking Neural Networks. *IEEE Signal Processing Magazine* **36,** 51–63 (2019).

2. Manis, P. B., Kasten, M. R. & Xie, R. Classification of neurons in the adult mouse cochlear nucleus: Linear discriminant analysis. *PLOS ONE* **14** (ed Malmierca, M. S.) e0223137 (10 2019).

3. Manis, P., Kasten, M. R. & Xie, R. *Raw voltage and current traces for current-voltage (IV) relationships for cochlear nucleus neurons.* 2019.

4. Lein, E. S. *et al.* Genome-wide atlas of gene expression in the adult mouse brain. *Nature* **445,** 168–176 (7124 2007).

5. Hawrylycz, M. J. *et al.* An anatomically comprehensive atlas of the adult human brain transcriptome. *Nature* **489,** 391–399 (7416 2012).

6. Cramer, B., Stradmann, Y., Schemmel, J. & Zenke, F. The heidelberg spiking datasets for the systematic evaluation of spiking neural networks. *IEEE Transactions on Neural Networks and Learning Systems,* 1–14 (2020).
